# Risk factors of severe fever with thrombocytopenia syndrome combined with central neurological complications: A five-year retrospective case–control study

**DOI:** 10.3389/fmicb.2022.1033946

**Published:** 2022-11-03

**Authors:** Min Wang, Peng Huang, Wei Liu, Weilong Tan, Tianyan Chen, Tian Zeng, Chuanlong Zhu, Jianguo Shao, Hong Xue, Jun Li, Ming Yue

**Affiliations:** ^1^Department of Infectious Diseases, The First Affiliated Hospital of Nanjing Medical University, Nanjing, China; ^2^Department of Epidemiology, Center for Global Health, School of Public Health, Nanjing Medical University, Nanjing, China; ^3^State Key Lab Pathogen and Biosecurity, Beijing Institute of Microbiology and Epidemiology, Beijing, China; ^4^Department of Infectious Disease Prevention and Control, Eastern Theater Command Centers for Disease Control and Prevention, Nanjing, China; ^5^Department Infectious and Tropical Diseases, The Second Affiliation Hospital of Hainan Medical University, Haikou, China; ^6^Department of Gastroenterology, Nantong Third People’s Hospital Affiliated to Nantong University, Nantong, China; ^7^Department of Hepatology, Nantong Third People’s Hospital Affiliated to Nantong University, Nantong, China

**Keywords:** central neurological complications, severe fever with thrombocytopenia syndrome, severe fever with thrombocytopenia syndrome virus, risk factors, retrospective study

## Abstract

**Objective:**

Severe fever with thrombocytopenia syndrome (SFTS) is an emerging infectious disease with high mortality rate, especially SFTS combined with central neurological complications. The purpose of this study was to explore risk factors of central neurological complications in SFTS patients.

**Methods:**

In this retrospective study, SFTS patients admitted to the First Affiliated Hospital of Nanjing Medical University between January 2017 and December 2021 were enrolled. Based on the presence or absence of central neurological complications, SFTS patients were divided into case group and control group. The patients’ laboratory parameters and clinical data were collected for statistical analysis. Receiver operating characteristic (ROC) curve analysis was used to evaluate the prediction accuracy of independent risk factors in identifying SFTS patients with central neurological complications.

**Results:**

In total, 198 hospitalized SFTS patients with complete medical records, clear etiological diagnosis and clinical outcomes were enrolled in this study. Of these, 74 (37.4%) cases were diagnosed with SFTS with central neurological complications, 29 (39.2%) cases died, and no death occurred in the control group. Multivariate logistic regression analysis revealed pulmonary rales, atrial fibrillation, and high serum SFTSV RNA, lactate dehydrogenase level during the fever stage as independent risk factors for the development of central neurological complications in SFTS patients. ROC curve analysis showed that the area under the ROC curve (AUC) of serum SFTSV RNA and lactate dehydrogenase levels were 0.748 (95%*CI*: 0.673–0.823, *p* < 0.001) and 0.864 (95%*CI*: 0.815–0.914, *p* < 0.001), respectively, in central neurological complications predicted in SFTS patients.

**Conclusion:**

Severe fever with thrombocytopenia syndrome (SFTS) combined with central neurological complications has high morbidity and mortality and diverse clinical manifestations. Early monitoring of lung signs, electrocardiogram, blood SFTSV RNA, and lactate dehydrogenase levels in SFTS patients may be useful in predicting the occurrence of central neurological complications.

## Introduction

Severe fever with thrombocytopenia syndrome (SFTS) is an acute infectious disease caused by Dabie bandavirus of the genus Bandavirus (formerly SFTS virus, SFTSV; Kuhn et al., 2020). This negative-strand RNA virus was first reported in rural Hubei and Henan provinces of China in 2009 (Yu et al., 2011). In the last 10 years, the number of SFTS cases and local outbreak areas has been increasing (Miao et al., 2020). Moreover, SFTS cases have been reported in several South-east Asian countries. Humans are generally susceptible to SFTSV, which can be transmitted through tick bites, or close contact with animals such as cats and dogs, as well as with SFTSV-infected people (Niu et al., 2013; Yoo et al., 2016; Jung et al., 2019; Kobayashi et al., 2020). SFTS clinically manifests as acute fever, gastrointestinal symptoms, low platelet (PLT), and white blood cell count (WBC), but these symptoms are usually self-limiting (Liu et al., 2014). At present, there is no unified standard for SFTS disease course. The most common method divides the clinical course of SFTS into three major stages: fever stage (the first week), multiple organ dysfunction stage (the second week), and recovery stage (2 weeks later; Liu et al., 2014). The fever stage and multiple organ dysfunction stage are the most critical stages in SFTS progression (Liu et al., 2014). Notably, some severe patients can rapidly progress to multiple organ dysfunction and even die. SFTS has a mortality rate of 10–30% (Li et al., 2018; Miao et al., 2020; Li et al., 2021). Currently, there is no available vaccine or specific antiviral drugs for SFTS, hence SFTS is one of the most important infectious diseases threatening public health.

Severe SFTS cases can present with central neurological complications, typically about 5 days after disease onset (Gai et al., 2012). The nervous system manifestations include apathy, dysphoria, muscular tremor, lethargy, confusion, coma, and convulsion (Gai et al., 2012; Li et al., 2018). Fatal outcomes have been associated with central neurological complications, with a higher case fatality rate reported when multiple neurological symptoms co-occur (Li et al., 2018). However, the pathogenesis and risk factors of central neurological complications induced by SFTSV are not yet known. A Korean study that retrospectively analyzed cerebrospinal fluids of eight patients with neurological symptoms found significantly higher monocyte chemoattractant protein-1 (MCP-1) and interleukin (IL)-8 levels in cerebrospinal fluid than in serum (Park et al., 2018). In addition, an autopsy study involving SFTS patient with hemophagocytic syndrome and neurological involvement found SFTSV nucleoprotein antigen-positive immunoblasts in multiple brain tissues (including the midbrain, pons, medulla, basal ganglia, cerebellum, and cerebrum) and the vascular lumina (Kaneko et al., 2018). It has been speculated that the central nervous system (CNS) damage in SFTS patients may be mediated by immune response. A retrospective study involving 121 SFTS patients in China found that subcutaneous bleeding, pulmonary rales, percentage of neutrophils, high lactate dehydrogenase and C-reactive protein levels, and low chloride ion concentration were strongly associated with the occurrence of central neurological complications (Fei et al., 2021). The significant decrease in chloride ion concentration 1–5 days after disease onset may be a useful risk factor for predicting the occurrence of central neurological complications in SFTS patients (Fei et al., 2021). Another Chinese retrospective study involving 109 SFTS patients identified bacteremia, procalcitonin > 0.5ug/l or serum amylase level > 80 U/l as independent predictors of SFTS progression with encephalopathy/encephalitis (Xu Y. et al., 2021). Obviously, SFTS fatal outcomes in SFTS patients are closely associated with central neurological complications (Gai et al., 2012; Gong et al., 2021). However, studies investigating the risk factors for SFTS combined with central neurological complications are fewer and their results are inconclusive. This study aimed to explore the risk factors of SFTS patients with central neurological complications by analyzing their clinical data to provide a basis for timely clinical intervention and treatment to reduce mortality.

## Materials and methods

### Study population

Initial data were collected from 259 hospitalized SFTS patients admitted to the First Affiliated Hospital of Nanjing Medical University (Jiangsu Provincial People’s Hospital) from January 2017 to December 2021. The diagnostic criteria used followed the 2010 edition of the Clinical Guidelines on Severe Fever with Thrombocytopenia Syndrome released by the Ministry of Health of the People’s Republic of China (Ministry of Health of People’s Repubic of China, 2010). Patients with incomplete case data and unclear clinical outcome were excluded. Then, according to the presence or absence of central neurological complications, patients were divided into two groups: patients with central neurological complications (case group or CNS) and the group without central neurological complications (control group or N).

Severe fever with thrombocytopenia syndrome (SFTS) diagnostic criteria: history of working, living, or traveling in mountainous and hilly areas during the epidemic season (from April to November), or having a history of being bitten by tick, or close contact with SFTSV-infected people; patients with acute onset, mainly manifesting as fever (armpit temperature above 38°C), accompanied by fatigue, anorexia, nausea, vomiting, etc.; low white blood cell count (< 4 × 10^9/l) and/or decreased platelet count (< 100 × 10^9/l); positive SFTSV RNA in peripheral blood (≥ 4.96 × 10^3 copies/ml).

The diagnostic criteria for SFTS combined with central neurological complications (Gai et al., 2012; Cui et al., 2015; Kato et al., 2016; Fei et al., 2021; Gong et al., 2021): patients who developed one or more of the following symptoms: paroxysmal involuntary muscle and limb tremor; personality and behavior changes, including irritability, apathy, dysphoria and unresponsiveness; cognitive impairment, including decline in orientation, memory and calculation, inability to accurately answer questions, inability to cooperate with simple commands, inability to perform fine exercise and aphasia, as well as language communication impairment; impaired consciousness, including drowsiness, lethargy, confusion, delirium, and coma; and convulsions or seizures.

The grouping criteria were according to the presence or absence of central neurological complications. Patients were divided into one group with central neurological complications (CNS) and another without central neurological complications (N).

SFTS clinical course is as follows: fever stage (the first week), multiple organ dysfunction stage (the second week) and recovery stage (2 weeks after onset). The fever stage and multiple organ dysfunction stage are the critical stages in SFTS progression (Liu et al., 2014) and also the onset period of central neurological complications (Gai et al., 2012).

### Clinical data collection

This study was a retrospective case–control study. Clinical data of the two patient groups, including age, gender, basic diseases, clinical manifestations, blood SFTSV RNA, blood routine, coagulation routine, biochemical indexes, electrocardiogram, chest CT, brain CT, and cerebrospinal fluid examination results after seeing doctors were collected. Blood samples were collected from SFTS patients every 1–2 days after admission and once a day for severe cases. In this study, laboratory results were compared between the two groups in fever stage (the first week) and multiple organ dysfunction stage (the second week). When multiple measurements were taken, the worst value were selected for the variable for each patient. Patients with repeated examination and follow-up were excluded from the data set finally analyzed. TaqMan real-time fluorescence quantitative polymerase chain reaction (PCR) kit (Da’an gene Co., Ltd., China) was used to quantitatively measure the viral load of SFTSV in serum and cerebrospinal fluid samples with a positive judgment standard ≥ 4.96 × 10^3 copies/ml (i.e., 3.7 lg copies/ml). The serum levels of IL-2, IL-4, IL-6, IL-10, tumor necrosis factor, and interferon-γ (IFN-γ) were tested using Beckman DxFLEX flow cytometer (Beckman Coulter, Inc., United States) and cytokine joint detection kit (Jiangxi Saiji Biotechnology Co., Ltd., China) following manufactures’ instructions.

### Statistical analysis

All statistical analysis were performed by SPSS 22.0 (SPSS Inc., Chicago, USA). Frequencies and percentages were used for categorical variables, whereas Chi-square test or Fisher’s exact test was used to identify differences between the two groups. Continuous variables with normal or non-normal distribution were presented as mean ± standard deviation (x ± s) or *M* (*Q25*, *Q75*). These variables were compared using the *t*-test or nonparametric test, respectively. Univariate and multivariate analyses were performed using binary logistic regression analysis. In multivariate analysis, a stepwise forward method was used to screen independent risk factors. ROC curve analysis was performed for the independent risk factors. In addition, AUC and the best cut-off value with the corresponding sensitivity and specificity were calculated to assess the predictive performance of the independent risk factors for SFTS with central neurological complications. *p* < 0.05 was considered statistically significant, whereas AUC > 0.7 was considered of clinical value.

## Results

### Demographic and clinical characteristics

**Figure 1:**
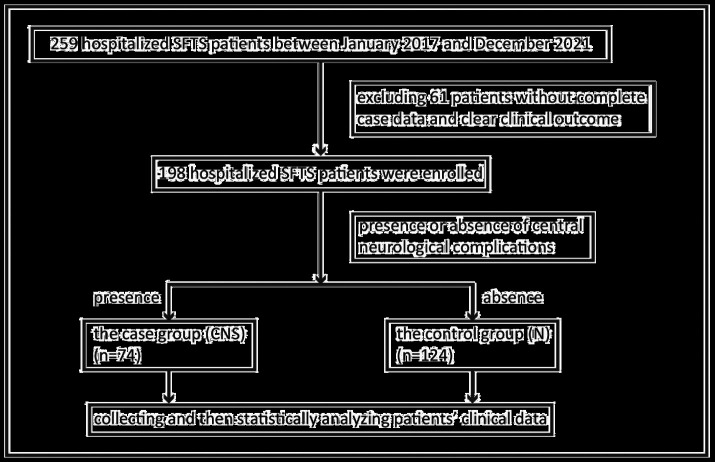
Schematic flow chart of SFTS patients combined with central neurological complications enrolment. SFTS, severe fever with thrombocytopenia syndrome.

Between January 2017 and December 2021, clinical data were initially collected from 259 hospitalized SFTS patients admitted to the First Affiliated Hospital of Nanjing Medical University (Jiangsu Provincial People’s Hospital). After excluding patients without complete case data and clear clinical outcome, 198 hospitalized SFTS patients were finally enrolled. The inclusion process of study objects is shown in Figure 1. Of the 198 patients, 74 (37.4%) cases were in the case group, comprising 39 males and 35 females with an average age of (65.4 ± 9.6) years and (67.5 ± 7.3) years, respectively. In addition, 124 (62.6%) cases were included in the control group, comprising 63 males and 61 females with a mean age of (57.3 ± 12.9) years and (59.4 ± 12.6) years, respectively. There was significant difference between the two groups in age (*p* < 0.05) but not in gender (Table 1).

**Table 1:** Comparison of clinical features and prognosis between the two groups.

	CNS (*n* = 74)	N (*n* = 124)	Statistic	*p*-value
*Demographic features*				
Age^a^ (y)	66.4 ± 8.6	58.4 ± 12.7	−5.265	<0.001
Gender^b^ (male/female)	39/35	63/61	0.067	0.796
*Days from onset to first visit at our hospital^d^ (d)*	5(4, 6)	5(4, 6)	−0.093	0.926
*Underlying diseases, n (%)*				
Hypertension^b^	22(29.7%)	25(20.2%)	4.193	0.123
Coronary heart disease^b^	1(1.4%)	2(1.6%)	0	1
Type 2 diabetes^b^	17(23.0%)	15(12.1%)	4.046	0.044
Old cerebral infarction^b^	35(47.3%)	17(13.7%)	26.997	<0.001
Chronic obstructive pulmonary diseases^b^	10(13.5%)	6(4.8%)	4.695	0.030
Chronic viral hepatitis^b^	11(14.9%)	10(8.1%)	2.260	0.133
Cancer^b^	3(4.1%)	5(4.0%)	0	1
*Clinical manifestation, n (%)*				
Fever^b^	73(98.6%)	124(100%)	1.684	0.194
Maximum body temperature^d^ (°C)	39(38.7, 39.5)	39(38.8, 39.6)	−0.306	0.760
Fatigue^b^	53(71.6%)	86(69.4%)	0.114	0.736
Headache^b^	14(18.9%)	28(22.6%)	0.372	0.542
Dizzy^b^	17(23.0%)	44(35.5%)	3.403	0.065
Myalgia^b^	18(24.3%)	38(30.6%)	0.913	0.339
Lymphadenopathy^b^	31(41.9%)	38(30.6%)	2.582	0.108
Anorexia^b^	28(37.8%)	48(38.7%)	0.015	0.903
Nausea^b^	16(21.6%)	41(33.1%)	2.960	0.085
Vomiting^b^	19(25.7%)	31(25.0%)	0.011	0.916
Diarrhea^b^	34(45.9%)	50(40.3%)	0.600	0.439
Cough^b^	25(33.8%)	26(21.0%)	3.981	0.046
Expectoration^b^	17(23.0%)	19(15.3%)	1.823	0.177
Petechiae/ecchymosis^b^	22(29.7%)	5(4.0%)	25.986	<0.001
Oral hemorrhage^b^	14(18.9%)	7(5.6%)	8.612	0.003
Hematemesis/melena^b^	12(16.2%)	4(3.2%)	8.852	0.003
Hemoptysis^b^	1(1.4%)	1(0.8%)	0	1
Pulmonary rale^b^	30(40.5%)	14(11.3%)	22.941	<0.001
Atrial fibrillation^b^	22(29.7%)	3(2.4%)	28.905	<0.001
Atrial/ventricular premature beats^b^	11(14.9%)	4(3.2%)	7.381	0.007
*Hospital mortality^c^*	29(39.2%)	0	–	<0.001

^a^By means of the *t*-test, expressed as mean ± standard deviation (*x* ± s); ^b^By means of the χ2 test, expressed by frequencies and percentages; ^c^By means of Fisher exact test; ^d^By means of the nonparametric test, expressed as *M* (*Q25*, *Q75*), *M* is the median, *Q25* is the lower quartile, *Q75* is the upper quartile. CNS, the case group; N, the control group; “–”: no relevant data.

The time from symptom onset to the first visit at our hospital was 1–7 days, a median time of 5 days. There was no significant difference between the two groups in days to their first visit at our hospital. After admission, all patients received ribavirin antiviral treatment, symptomatic support and organ function protection treatment. Those with body temperature ≥ 39°C, WBC < 2.0 × 10^9/l, PLT < 30 × 10^9/l received glucocorticoid, subcutaneous injection of recombinant human granulocyte colony stimulating factor, and platelet transfusion, respectively, whereas those with activated partial thromboplastin time (APTT) > 60s were infused with fresh plasma and cryoprecipitate. Severe patients and those with bacterial or fungal infection were, respectively, injected with gamma globulin intravenously and antibiotics intravenously. In total, 29 (14.6%) of 198 patients, all in the case group, died (Table 1).

Regarding underlying diseases, compared with the control group, the case group had a higher prevalence of type 2 diabetes, old cerebral infarction and chronic obstructive pulmonary disease (all *p* < 0.05; Table 1).
Regarding clinical manifestations, compared with the control group, the incidence of cough, pulmonary rales, petechiae/ecchymosis, oral hemorrhage, hematemesis/melena, atrial fibrillation, and atrial/ventricular premature beats was higher in the case group (all *p* < 0.05; Table 1).

### Laboratory parameters comparison

During the fever stage (the first week), the levels of blood SFTSV load, prothrombin time (PT), APTT, thrombin time (TT), D-dimer, alanine aminotransferase (ALT), aspartate aminotransferase (AST), blood urea nitrogen (BUN), creatinine (Cr), lactate dehydrogenase (LDH), creatine kinase (CK), α-hydroxybutyrate dehydrogenase (α-HBDH), and potassium in the case group were higher than those in the control group (all *p* < 0.05). Contrastingly, levels of PLT and calcium in the case group were lower than those in the control group (all *p* < 0.05; Table 2). Laboratory parameter comparison between the two groups in the multiple organ dysfunction stage (the second week) is shown in Supplementary Table S1. During the multiple organ dysfunction stage, some indicators (including PT, APTT, TT, LDH, CK, α-HBDH, etc.) were not reexamined due to the death or improvement of some patients. SFTSV RNA was also not reexamined in majority of the surviving patients. Consequently, only 138 patients, comprising 64 cases in the case group and 74 cases in the control group, were finally enrolled and SFTSV RNA was not included in the analysis. The WBC count, levels of PT, APTT, TT, D-Dimer, AST, BUN, Cr, LDH, CK, and α-HBDH were higher in the case group than in the control group (all *p* < 0.05), whereas PLT count was lower in the case group than in the control group (*p* < 0.05) as shown in Supplementary Table S1.

**Table 2:** Comparison of laboratory parameters between the two groups during the fever stage.

	CNS (*n* = 74)	N (*n* = 124)	Statistic	*p*-value
SFTSV RNA^a^ (lg copies/ml)	6.66 ± 1.45	5.50 ± 0.99	−6.080	<0.001
WBC^b^(× 10^9/l)	1.98(1.21, 2.73)	1.81(1.28, 2.24)	−0.747	0.455
PLT^b^(×10^9/l)	36(27, 52)	50(37, 73)	−4.191	<0.001
PT^b^(s)	12.4(11.7, 13.2)	12.2(11.6, 12.9)	−2.284	0.022
APTT^b^(s)	48.3(42.6, 59.7)	41.0(35.4, 47.4)	−5.513	<0.001
TT^b^(s)	32.0(22.6, 73.6)	22.0(19.8, 25.2)	−6.023	<0.001
D-dimer^b^(mg/l)	4.72(2.64, 10.39)	2.18(1.20, 4.78)	−4.192	<0.001
ALT^b^(U/l)	99.0(56.2, 179.5)	67.5(46.9, 123.5)	−2.875	0.004
AST^b^(U/l)	394.3(157.8, 629.1)	160.4(92.4, 317.3)	−5.330	<0.001
BUN^b^(mmol/l)	7.09(5.45, 10.19)	5.63(4.44, 7.52)	−3.673	<0.001
Cr^b^(μmol/l)	80.0(62.8, 104.3)	70.6(58.1, 87.9)	−2.796	0.005
LDH^b^(U/l)	1,499(1,075, 2,420)	708(471, 1,004)	−8.448	<0.001
CK^b^(U/l)	1,073(409, 1,593)	339(134, 835)	−5.886	<0.001
α-HBDH^b^(U/l)	707(467, 1,078)	363(254, 524)	−7.072	<0.001
K^a^(mmol/l)	3.92 ± 0.46	3.76 ± 0.49	−2.233	0.027
Na^a^(mmol/l)	133.2 ± 4.9	134.3 ± 3.8	1.791	0.075
Ca^a^(mmol/l)	1.98 ± 0.14	2.04 ± 0.13	3.362	0.001

^a^By means of the *t*-test, expressed as mean ± standard deviation (x ± s); ^b^By means of the nonparametric test, expressed as *M* (*Q25* and *Q75*), *M* is the median, *Q25* is the lower quartile, *Q75* is the upper quartile. CNS, the case group; N, the control group; SFTSV, severe fever with thrombocytopenia syndrome virus; WBC, white blood cell; PLT, platelet; PT, prothrombin time; APTT, activated partial thromboplastin time; TT, thrombin time; ALT, alanine aminotransferase; AST, aspartate aminotransferase; BUN, blood urea nitrogen; Cr, creatinine; LDH, lactate dehydrogenase; CK, creatine kinase; α-HBDH, α-hydroxybutyrate dehydrogenase.

### Central neurological symptoms and clinical data of cerebrospinal fluid testing

**Figure 2:**
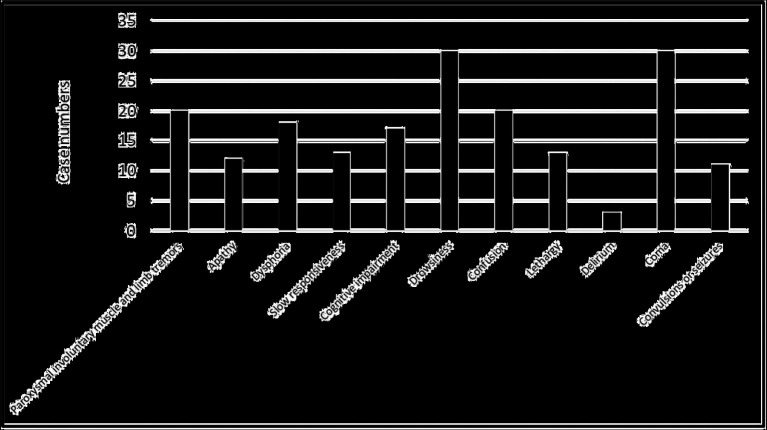
Incidence of central nervous system symptoms in the case group.

Central nervous system symptoms of patients in the case group occurred within 1–14 days after disease onset, averaging 6 days. Among these patients, 20 (27.0%) had paroxysmal involuntary muscle and limb tremors, 12 (16.2%) had apathy, 18 (24.3%) had dysphoria, 13 (17.6%) had slow responsiveness, 17 (23.0%) had cognitive impairment, 30 (40.5%) had drowsiness, 20 (27.0%) had confusion, 13 (17.6%) had lethargy, 3 (4.1%) had delirium, 30 (40.5%) had coma, and 11 (14.9%) had convulsions or seizures, as shown in Figure 2.

In addition, 16 patients in the case group had performed lumbar puncture and cerebrospinal fluid testing at different times of the disease course. The cerebrospinal fluid-SFTSV-RNA was detected in nine cases, with only one (11.1%) case showing positive result for SFTSV. Cerebrospinal fluid-SFTSV-RNA and blood-SFTSV-RNA were simultaneously detected in six cases. Of these, only one case showed negative results for both cerebrospinal fluid-SFTSV-RNA and blood-SFTSV-RNA. Further, the remaining five cases showed lower cerebrospinal fluid-SFTSV-RNA than blood-SFTSV-RNA. Of the 16 patients, three cases had elevated white blood cell count and seven cases had red blood cell in cerebrospinal fluid. The protein level in cerebrospinal fluid went up in 15 cases, but remained within normal range in one case. In addition, glucose was elevated in the cerebrospinal fluid in 12 cases but remained within normal range in four cases. Lastly, cerebrospinal fluid chlorine was elevated in four cases, all of which died (Table 3).

**Table 3:** Cerebrospinal fluid testing of 16 patients in the case group.

Case no.	Age/Gender	Days of onset	Cerebrospinal fluid-SFTSV-RNA (lg copies/ml)	Blood-SFTSV-RNA (lg copies/ml)	WBC (×10^6/l)	RBC (×10^6/l)	Protein (g/l)	Glucose (mmol/l)	Chlorine (mmol/l)	Brain CT	Outcome
Normal range			<3.7	<3.7	0–8	0	0.2–0.4	2.5–4.4	120–130		
1	72/M	9th	Negative	NA	1	0	0.8↑	5.98↑	133.2↑	Lacunar infarction	Died
2	66/F	8th	Negative	5.76	2	0	0.5↑	6.1↑	130.5↑	Lacunar infarction	Died
3	76/F	7th	Negative	7.04	2	0	0.7↑	9.09↑	123.9	NA	Died
4	67/M	17th	Negative	Negative	8	0	0.4	6.92↑	133.7↑	Lacunar infarction	Died
5	70/F	15th	Negative	NA	2	0	0.7↑	6.39↑	128.2	Lacunar infarction	Survived
6	61/M	8th	Negative	4.9	1	0	1.1↑	7.02↑	128	Lacunar infarction	Died
7	68/M	11th	NA	NA	1	200↑	0.58↑	2.92	119.2↓	Lacunar infarction	Survived
8	53/M	10th	NA	4.92	2	20↑	0.6↑	6.88↑	129.2	Cerebral infarction	Survived
9	43/M	14th	NA	Negative	10↑	200↑	1.16↑	2.93	121.1	Lacunar infarction	Survived
10	71/F	9th	NA	3.94	5	60↑	0.69↑	4.76↑	121.4	Lacunar infarction	Survived
11	67/M	10th	Negative	5.68	13↑	0	1.4↑	8.03↑	129	NA	Survived
12	74/F	8th	NA	9.28	1	0	0.7↑	6.71↑	125.3	Lacunar infarction	Survived
13	67/F	8th	NA	4.92	42↑	36↑	1.6↑	3.78	124.1	Lacunar infarction	Survived
14	64/F	13th	4.63	8.34	0	8↑	1.06↑	10.2↑	147.7↑	NA	Died
15	61/F	7th	NA	9.09	3	10↑	0.83↑	8.01↑	125.9	Lacunar infarction	Died
16	66/M	14th	Negative	NA	2	0	0.88↑	3.36	127.3	Lacunar infarction	Survived

NA, not available; 1 lg copies/ml = 10^1 copies/ml. SFTSV, severe fever with thrombocytopenia syndrome virus; WBC, white blood cell; RBC, red blood cell.

### Univariate and multivariate logistic regression analysis of risk factors of SFTS combined with central neurological complications

Laboratory parameters during the fever stage combined with the clinical parameters in the disease course were analyzed to identify the risk factors of SFTS with CNS complications at an early stage. Univariate logistic regression analysis showed that age, type 2 diabetes, old cerebral infarction, chronic obstructive pulmonary disease, cough, pulmonary rales, petechiae/ecchymosis, oral hemorrhage, hematemesis/melena, atrial fibrillation, atrial/ventricular premature beats, as well as high-level SFTSV load, PT, APTT, TT, ALT, AST, BUN, Cr, LDH, CK, α-HBDH, K, and low-level PLT and Ca during the fever stage were the risk factors of central neurological complications in SFTS patients. Multivariate stepwise forward regression analysis was performed on the risk factors with statistically significant differences from the univariate analysis. The results showed that pulmonary rales, atrial fibrillation, and high-level SFTSV load and high-level LDH during the fever stage were independent risk factors predicting central neurological complications in SFTS patients (Table 4).

**Table 4:** Univariate and multivariate logistic regression analysis of SFTS patients with central neurological complications.

	Univariate			Multivariate		
	*OR*	*p*-value	95%*CI*	*OR*	*p*-value	95%*CI*
Age	**1.072**	**<0.001**	1.039, 1.106			
Type 2 diabetes	**2.167**	**0.047**	1.009, 4.656			
Old cerebral infarction	**5.649**	**<0.001**	2.846, 11.213			
Chronic obstructive pulmonary disease	**3.073**	**0.037**	1.068, 8.842			
Cough	**1.923**	**0.048**	1.007, 3.674			
Pulmonary rales	**5.357**	**<0.001**	2.596, 11.054	**4.931**	**0.001**	1.892, 12.851
Petechiae/ecchymosis	**10.069**	**<0.001**	3.616, 28.042			
Oral hemorrhage	**3.900**	**0.005**	1.495, 10.177			
Hematemesis/melena	**5.806**	**0.003**	1.798, 18.753			
Atrial fibrillation	**17.064**	**<0.001**	4.892, 59.517	**21.598**	**<0.001**	4.896, 95.270
Atrial/ventricular premature beats	**5.238**	**0.006**	1.603, 17.120			
SFTSV RNA	**2.297**	**<0.001**	1.697, 3.109	**1.826**	**0.005**	1.194, 2.792
PLT	**0.979**	**0.002**	0.966, 0.992			
PT	**1.235**	**0.042**	1.007, 1.514			
APTT	**1.076**	**<0.001**	1.044, 1.108			
TT	**1.026**	**<0.001**	1.014, 1.039			
D-Dimer	1.017	0.172	0.993, 1.043			
ALT	**1.005**	**0.005**	1.001, 1.008			
AST	**1.003**	**<0.001**	1.002, 1.004			
BUN	**1.190**	**<0.001**	1.082, 1.309			
Cr	**1.012**	**0.005**	1.004, 1.021			
LDH	**1.002**	**<0.001**	1.001, 1.003	**1.002**	**<0.001**	1.001, 1.002
CK	**1.001**	**<0.001**	1.000, 1.001			
α-HBDH	**1.004**	**<0.001**	1.002, 1.005			
K	**1.996**	**0.029**	1.074, 3.712			
Ca	**0.027**	**0.001**	0.003, 0.245			

Statistically significant results were shown in bold. SFTS, severe fever with thrombocytopenia syndrome; SFTSV, severe fever with thrombocytopenia syndrome virus; PLT, platelet; PT, prothrombin time; APTT, activated partial thromboplastin time; TT, thrombin time; ALT, alanine aminotransferase; AST, aspartate aminotransferase; BUN, blood urea nitrogen; Cr, creatinine; LDH, lactate dehydrogenase; CK, creatine kinase; α-HBDH, α-hydroxybutyrate dehydrogenase; *OR*, odds ratio; 95% *CI*: 95% confidence interval.

### Receiver operating characteristic curve analysis of serum SFTSV RNA and LDH during the fever stage in SFTS patients combined with central neurological complications

**Figure 3:**
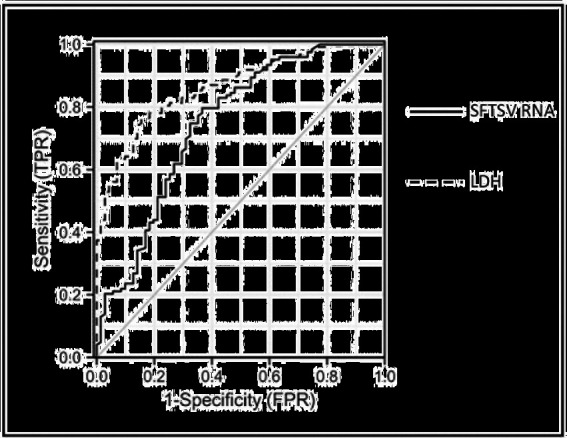
Receiver operating characteristic curve analysis of serum SFTSV RNA and LDH during the fever stage in SFTS patients combined with central neurological complications^*^. ^*^The data of 194 patients (71 cases in case group and 123 cases in control group) were used to assess the predictive value of serum SFTSV RNA and LDH during the fever stage in SFTS patients with central neurological complications. ROC, receiver operating characteristic; SFTS, severe fever with thrombocytopenia syndrome; SFTSV, severe fever with thrombocytopenia syndrome virus; LDH, lactate dehydrogenase.

Laboratory parameters during the fever stage were analyzed and serum SFTSV RNA and LDH were identified as independent risk factors for SFTS combined with CNS complications. Further ROC curve analysis was performed to search biomarkers for predicting SFTS with CNS complications. As shown in Figure 3 and Table 5, ROC curve analysis of serum SFTSV RNA during the fever stage yielded an AUC of 0.748 (95%*CI*: 0.673–0.823, *p* < 0.001) with the youden index of 0.431 for predicting SFTS patients combined with central neurological complications. At a cut-off value of 6.21 lg copies/ml (about 1.62 × 10^6 copies/ml), serum SFTSV RNA had a sensitivity of 79.7% and a specificity of 63.4%. Similarly, ROC curve analysis of serum LDH during the fever stage yielded an AUC of 0.864 (95%*CI*: 0.815–0.914, *p* < 0.001) with the youden index of 0.609 for predicting SFTS patients combined with central neurological complications. Further, at a cut-off value of 990.00 U/l, serum LDH had a sensitivity of 76.4% and a specificity of 84.5%.

**Table 5:** Parameters related to ROC curve of serum SFTSV RNA and LDH during the fever stage in SFTS patients with central neurological complications^*^.

	Sensitivity	Specificity	Youden index	*Cut-off* value	AUC (95%*CI*)	*p*-value
SFTSV RNA (lg copies/ml)	0.797	0.634	0.431	6.210	0.748(0.673–0.823)	<0.001
LDH (U/l)	0.764	0.845	0.609	990.00	0.864(0.815–0.914)	<0.001

1 lg copies/ml = 10^1 copies/ml. ^*^The data of 194 patients (71 cases in case group and 123 cases in control group) were used to assess the predictive value of serum SFTSV RNA and LDH during the fever stage in SFTS patients with central neurological complications. ROC, receiver operating characteristic; AUC, area under the ROC curve; SFTS, severe fever with thrombocytopenia syndrome; SFTSV, severe fever with thrombocytopenia syndrome virus; LDH, lactate dehydrogenase; 95% *CI*: 95% confidence interval.

## Discussion

In this study, of the 198 hospitalized patients with SFTS, 74 (37.4%) developed central neurological complications and 29 (39.2%) of them died. Multivariate logistic regression analysis identified pulmonary rales, atrial fibrillation, as well as high-level serum SFTSV load and LDH during the fever stage as independent risk factors of central neurological complications for SFTS patients. Moreover, ROC curve analysis showed that the AUC of serum SFTSV RNA and LDH predicting the occurrence of CNS complications in SFTS patients were 0.748 (95%*CI*: 0.673–0.823, *p* < 0.001) and 0.864 (95%*CI*: 0.815–0.914, *p* < 0.001), respectively.

Previous studies have reported an incidence rate of central neurological complications in SFTS patients ranging from 19.1 to 76% (Cui et al., 2015; Li et al., 2018; Park et al., 2018; Gong et al., 2021), consistent with the result of this study (37.4%). Cui et al. (2015) reported that 19.1% of SFTS patients developed central neurological complications, which manifested as blurred mind, dysphoria, convulsion, drowsiness, and coma, with 44.7% of them dying. In the present study, central neurological complications were associated with a comparatively higher morbidity but lower mortality, possibly due to the different diagnostic criteria for central neurological complications in these two studies. SFTS patients can develop CNS symptoms such as muscle and limb tremor, dysphoria, apathy, slow responsiveness, and disorientation, which have also been reported in several studies, and all these symptoms often precede serious impairment of consciousness (Gai et al., 2012; Cui et al., 2015; Kato et al., 2016). Therefore, we also included patients with paroxysmal involuntary muscle and limb tremor, personality and behavior changes (such as apathy, slow responsiveness, etc.) and cognitive impairment in the case group purposely to discover CNS damage as early as possible and achieve timely diagnosis.

In addition, patients in the case group were more likely to experience respiratory symptoms, bleeding and arrhythmia, which was consistent with the results of Fei et al.’s study (Fei et al., 2021). After multivariate logistic regression analysis, pulmonary rales, atrial fibrillation were identified as independent risk factors for SFTS with central neurological complications. Thus, observing lung signs and improving electrocardiogram as soon as possible may help in timely diagnosis of patients suspected of having CNS damage. Scholars have reported a SFTS case with reversible myocardial dysfunction and encephalopathy, who experienced impaired consciousness, Kernig sign and myoclonus to face and limbs, and cardiomegaly and abnormal T waves as shown by Chest X-ray and electrocardiography, respectively (Kawaguchi et al., 2016). Another study reported consciousness disorder and fulminant myocarditis in a SFTS patient whose electrocardiogram also showed diffuse ST elevation (Miyamoto et al., 2019). Thus, it is not uncommon for SFTS patients with central neurological complications to experience myocardial dysfunction and diverse electrocardiography manifestations, although the pathogenesis is still not well understood. It is known that cardiomyocytes could be damaged by viruses directly or indirectly (immune response) (Yajima, 2011), but no evidence of SFTSV replication in heart parenchymal cells has been noted in any autopsy reports (Nakamura et al., 2019; Iwao et al., 2021). Patients with SFTS complicated with CNS damage often have hypercytokinemia (Cui et al., 2015; Kaneko et al., 2018), an immune reaction that is speculated to cause myocardial dysfunction in these patients. Moreover, clinical studies also found that acute viral encephalitis could produce various changes in electrocardiography possibly because of sympathetic discharge triggered by an increase in intracranial pressure caused by intracranial lesions (Magnano et al., 2002; Huemer et al., 2012). In addition, alterations in autonomic nervous system (composed of sympathetic nervous system, parasympathetic nervous system, and intestinal nervous system) have been linked to variations in cardiac repolarization and high risk of malignant arrhythmia and cardiac arrest (Magnano et al., 2002). In the current study, cardiac rhythm abnormalities may be associated with CNS damage, but the manifestations were diverse. Future studies with larger samples are need to reveal the relationship between CNS damage and myocardial dysfunction in SFTS patients.

Through multivariate logistic regression analysis, we also found that high-level serum SFTSV RNA was an independent risk factor for central neurological complications in SFTS patients. The ROC curve analysis showed that serum SFTSV RNA had a good predictive value on CNS complications in SFTS patients (AUC = 0.748). Firstly, viruses can enter the CNS *via* the blood cerebrospinal fluid barrier, which is composed of choroid plexus epithelial cells (Schwerk et al., 2015). Access of some viruses to the CNS *via* their receptors has been described. For example, Coxsackievirus B3 could enter the cerebrospinal fluid by binding to coxsackie and adenovirus receptor located in the tight junction between choroid plexus epithelial cells (Tabor-Godwin et al., 2010). By comparison, SARS-COV-2 disrupted the blood-cerebrospinal fluid-barrier in human brain organoids by binding with ACE2 receptor expressed on choroid plexus epithelial cells (Pellegrini et al., 2020). Although no SFTSV receptor had been reported or identified on human neuron cell at present and no evidence of direct SFTSV infection of brain parenchymal cells has been found in any autopsy reports (Hiraki et al., 2014; Uehara et al., 2016; Kaneko et al., 2018; Nakamura et al., 2019; Iwao et al., 2021), several studies have found SFTSV in CNS. SFTSV has been detected in cerebrospinal fluid of SFTS patients combined with central neurological complications (Cui et al., 2015; Park et al., 2018). Cui et al. (2015) inoculated the cerebrospinal fluid sample from SFTS-related human encephalitis survivor on the 8th day after admission into DH82 cells, successfully cultured and isolated one SFTSV strain (HNXY-319). In addition, SFTSV RNA was detected simultaneously in serum and cerebrospinal fluid using real-time quantitative PCR (serum: 4.47 × 10^3 copies/ml, cerebrospinal fluid: 4.68 × 10^3 copies/ml) (Cui et al., 2015). Park et al. (2018) measured SFTSV RNA in cerebrospinal fluid of eight SFTS patients with suspected SFTS-associated encephalopathy/encephalitis on the 3rd–7th day of the disease course using TaqMan probe real-time PCR. Of the eight patients, six (75%) showed positive results for SFTSV (Park et al., 2018). SFTSV loads in cerebrospinal fluid (range: 2.0 × 10^3–6.0 × 10^6 copies/ml) were significantly lower than those in serum (range: 1.6 × 10^6–3.2 × 10^11 copies/ml) (Park et al., 2018). In this study, SFTSV RNA was measured in cerebrospinal fluid samples using the same method in nine patients in the case group between the 7th and 17th day of the disease course (i.e., on the 9th, 8th, 7th, 17th, 15th, 8th, 10th, 13th, and 14th days). Of the nine patients, eight (88.9%) showed negative results for SFTSV, and only one (11.1%) showed positive result for SFTSV (4.22 × 10^4 copies/ml) on the 13th day of the disease course. The positive rate of SFTSV RNA in cerebrospinal fluid in this study was significantly lower than that reported by Park et al. (2018), which may be due to the different examination time or test reagents used in both studies.

Secondly, brain microvascular endothelial cells as well as the intercellular tight junction, pericytes, astrocytes, and extracellular matrix are components of the blood–brain barrier (Obermeier et al., 2013). Impairment in any of these components may lead to the destruction of the blood–brain barrier (Obermeier et al., 2013). Some viruses such as Japanese encephalitis virus and West Nile virus can directly invade host endothelial cells to cross the blood–brain barrier by transcellular migration (Liou and Hsu, 1998; Verma et al., 2009). In contrast, other viruses such as dengue virus can invade tight junctions formed by endothelial cells of the blood–brain barrier to enter the CNS *via* paracellular migration (Velandia-Romero et al., 2016). Moreover, viruses such as West Nile virus, Nipah virus, Hepatitis C virus, dengue virus can hijack infected host immune cells (such as neutrophils and macrophages) to cross the blood–brain barrier through tight junctions and then release the virus, that is, penetrate into CNS using the “Trojan horse” strategy (Verma et al., 2009; Weissenborn et al., 2009; Velandia-Romero et al., 2016; Paul et al., 2017; Tiong et al., 2018). This refers to a strategy in which infected phagocytes such as monocytes, macrophages, or neutrophils act as “Trojan horses” to migrate and invade host organs and tissues, including CNS, to spread pathogens, including bacteria, fungi, viruses, etc. (Kim, 2008; Zhu et al., 2020; Strickland and Shi, 2021). Remarkably, viruses may utilize not only a single but a combination of these mechanisms to enter CNS (Suen et al., 2014). At present, SFTSV has been proved to mediate vascular endothelial injury and vascular barrier damage, which is positively associated with SFTSV load (Li et al., 2017; Xu S. et al., 2021). An autopsy study of a fatal SFTS patient with neurological involvement reported elevated serum level of SFTSV load before death (Kaneko et al., 2018). The highest SFTSV level of 1.41 × 10^9 copies/ml was reached on the 11th day of the disease course as detected using RT-PCR (Kaneko et al., 2018). This patient died on the 13th day from onset of the first symptom. Focal neuronal cell degeneration of the pons, infiltration of SFTSV-nucleocapsid protein-positive immunoblasts in the various brain tissues and their vascular lumina, hemosiderin-laden macrophages around extended microvessels, perivascular inflammatory cells infiltration and intravascular fibrin deposition were observed on autopsy, indicating vascular endothelial injury with inflammation (Kaneko et al., 2018). It was speculated that SFTSV may enter CNS through the damaged blood–brain barrier and invade brain tissues using the “Trojan horse” strategy. In this study, in addition to significantly higher levels of serum SFTSV RNA observed in the case group, higher incidence of petechiae/plaque, oral bleeding, hematemesis/melena were also found in the case group. This could be due to the coagulation dysfunction caused by thrombocytopenia and the destruction of vascular endothelial integrity (Xu S. et al., 2021), suggesting that the latter may be associated with the complications caused by SFTSV invasion of the CNS. However, more mechanistic studies are needed to unravel this relationship.

Evidence has shown that high serum SFTSV load can induce unbalanced cytokine distribution in the host’s body (including in CNS tissues) which causes immunopathological damage to the CNS (Sun et al., 2012; Liu et al., 2016). High levels of SFTSV RNA, interferon gamma-induced protein-10, IFN-γ, IL-8, and MCP-1 were detected in the serum from a SFTS patient with hemophagocytic syndrome and neurological damage before death (at 8–12 days of during the course of disease) (Kaneko et al., 2018). Similarly, SFTS patients who experienced encephalitis showed elevated serum SFTSV loads on day 7 after the disease onset (Cui et al., 2015). In addition, blood eosinophil leukocyte chemotactic factor, IFN-γ, IL-15, IL-6, interferon gamma-induced protein-10, and tumor necrosis factor-α were significantly elevated before disease deterioration in a non-fatal encephalitis SFTS patient, which may be due to encephalitis development (Cui et al., 2015). The levels of MCP-1 and IL-8 were significantly higher in cerebrospinal fluid compared with serum from patients with SFTS-associated encephalitis/encephalopathy collected between 3–7 days of the disease course (Park et al., 2018). In this study, immunofluorescence assay was performed to measure serum levels of cytokines (including IL-2, IL-4, IL-6, IL-10, tumor necrosis factor, and IFN-γ) for three cases in the case group and two cases in the control group during the fever stage, 10 cases in the case group and 11 cases in the control group during the multiple organ dysfunction stage. Results showed that serum levels of IL-10 in patients from the case group were found significantly higher compared with levels in the control group during the multiple organ dysfunction stage (Supplementary Tables S2, S3). It has been reported that serum levels of IL-6 and IL-10 are increased in severe viral infection. Within COVID-19 patients, serum IL-6 and IL-10 levels, which were found to be predictive of disease severity, are significantly higher in critical group than in moderate and severe group (Han et al., 2020). de Jong et al. (2006) obtained throat and blood specimens in influenza H5N1 infection in parallel on the same day, and found that serum interferon gamma-induced protein-10, MCP-1, monokine induced by interferon-gamma, IL-8, IFN-γ, IL-6, and IL-10 levels in H5N1 patients were significantly higher than in healthy controls, of which interferon gamma-induced protein 10, MCP-1, IL-8, IL-6 and IL-10 levels were positively correlated with pharyngeal H5N1 load, indicating that hypercytokinemia and hyperchemokinemia reflect may increase viral replication. You et al. showed that elevated serum IL-6, and IL-10 levels in patients with an SFTSV infection in South Korea strongly correlated with death outcomes (Yoo et al., 2021). However, there was no significant difference in serum IL-6 level between the two groups in our study, which may be due to the small sample size. IL-10 is a crucial anti-inflammatory cytokine that protects the host from own tissue damage by limiting excessive immune response to pathogens (Saraiva et al., 2020). However, this inhibitory property is also exploited by viruses to escape immune attack (Brooks et al., 2006, 2008). Choi et al. (2019) confirmed that the non-structural protein of SFTSV targeted the tumor progression locus 2 signaling pathway to induce IL-10 production and finally promote virus replication. In the study by Song et al. (2018), it was found that IL-10 disrupted the B cell-mediated immunity by suppressing germinal center formation and inhibiting the differentiation of dendritic cells. It is likely that the SFTSV hijacks some cytokines thereby promoting CNS complications in SFTS patients. In this study, data on serum cytokine levels was only available for 26 patients. Further studies with large sample sizes are needed to explore the associated mechanism.

The brain microvascular endothelial cell injury, inflammatory cell infiltration in brain tissue, and cytokine disorder mediated by SFTSV may contribute to SFTS-associated CNS damage. Therefore, serum SFTSV RNA and cytokine levels should be dynamically monitored in SFTS patients with active viral replication to allow timely initiation of therapeutic intervention and avoid CNS damage and adverse outcomes. Further studied are needed to examine the pathogenesis of CNS complications in SFTS patients.

Our results showed that serum LDH is an independent risk factor of SFTS associated with central neurological complications. Moreover, serum LDH was found to be a good predictor of CNS complications in SFTS patients (AUC = 0.846). LDH is an intracellular enzyme involved in anaerobic glycolysis. This enzyme is expressed in almost all organs and it catalyzes the mutual transformation of pyruvate and lactic acid. The release of this enzyme into peripheral blood is triggered by damage to cells. Therefore, LDH has been proposed to be a marker of cell injury (Ding et al., 2017; Guo et al., 2021). LDH is composed of two major subunits, LDH-A, and LDH-B, which assemble into five isoenzymes (LDH1, LDH2, LDH3, LDH4, and LDH5) (Guo et al., 2021). LDH1 is mainly expressed in cardiomyocytes, LDH2 in reticuloendothelial system, LDH3 in pneumocytes, LDH4 in the kidney, and LDH5 in the liver and striated muscle (Henry et al., 2020). The serum LDH levels detected in routine clinical tests is the total LDH level, and not the level of each isoenzyme. Studies have shown that SFTSV is a tropic virus which can invade various human tissue cells to induce the production of cytokines resulting in tissue cell damage and LDH release (Sun et al., 2012). Abnormally elevated serum LDH level in SFTS patients with CNS damage may reflect tissue and cell damage or even organ failure, but the correlation between different LDH isoenzymes and CNS damage has not been clarified. A recent study involving 144 patients with COVID-19 found that the serum LDH level was associated with COVID-19 severity (Yan et al., 2021). Further isozyme examination revealed that LDH2, LDH4, and LDH5 were significantly increased in patients with severe disease (Yan et al., 2021). In addition, it was found that the increase in serum LDH levels may be a result of inflammation-related tissue damage and hypoxia-related metabolic abnormalities through proteomics and metabolomics research (Yan et al., 2021). Understanding the mechanism underlying the elevation of LDH level in SFTS patients may reveal the correlation between different LDH types and disease course. This will improve the treatment and prognosis of SFTS patients.

Previously, some studies characterized the cerebrospinal fluid from SFTS patients with central neurological complications. For instance, Cui et al. (2015) found that the protein content and glucose levels were increased in the cerebrospinal fluid of two SFTS patients with encephalitis (a non-fatal patient and a fatal patient). In contrast, Park et al. revealed that the protein content and glucose levels in cerebrospinal fluid of 14 SFTS patients with suspected encephalitis/encephalopathy (12 survivors and 2 dead patients) were normal, and cerebrospinal fluid pleocytosis was rare (4/14) (Park et al., 2018). In this study, 16 patients (including 9 surviving patients and 7 dead patients) in the case group underwent cerebrospinal fluid testing. Cerebrospinal fluid pleocytosis (3/16) was also rare, but the levels of protein (15/16) and glucose (13/16) in cerebrospinal fluid were elevated. Moreover, of the 16 patients, 4 patients with elevated chloride died, and this has not been reported previously. Therefore, examination of cerebrospinal fluid should be performed as early as possible for patients with neurological symptoms to determine the existence of central involvement.

Underlying chronic diseases may play contribute to the pathogenicity of viral infection. A previous study assessed the correlation between SFTS clinical outcomes and multiple comorbidities. They found that patients complicated with diabetes mellitus, chronic viral hepatitis, and chronic obstructive pulmonary disease tended to have fatal outcomes, probably due to immune disorders (Zhang et al., 2019). In our study, patients complicated with type 2 diabetes and chronic obstructive pulmonary disease were at a higher risk of developing central neurological complications. In addition, we found for the first time that the prevalence of old cerebral infarction (mainly lacunar infarction) in the case group was significantly higher compared with that of the control group. Studies have shown that endothelial dysfunction and blood–brain barrier destruction are the main processes contributing to the pathogenesis of lacunar cerebral infarction (Caplan, 2015). Old brain damage in patients with lacunar infarction may increase the spread of SFTSV to the CNS causing neurological symptoms. However, 20–50% of healthy elderly people have been reported to have silent lacunar cerebral infarction (Vermeer et al., 2007). In this study, brain lacunar cerebral infarction in the case group was diagnosed by brain CT. The higher prevalence of lacunar cerebral infarction in the case group could be a result of the more brain CT performed on patients because of the occurrence of neurological symptoms. Therefore, large-scale well-designed studies are needed to validate this finding.

This study had several limitations. First, this was a single-center study and only five-year patients were enrolled, hence there could be some degree of selection bias. Second, the analyzed sample size was small and laboratory data with missing value of more than 10% (especially in the multiple organ dysfunction stage) were not included in the analysis, the statistical strength may be limited. The results of some observation indicators are listed in the Supplementary material for reference. Third, the pathogenesis of CNS complications in SFTS patients was not investigated in this study. Therefore, future prospective studies involving a large number of specimens from multiple centers should be conducted to identify the risk factors and mechanisms driving the development of central neurological complications in SFTS patients.

## Conclusion

This study found that SFTS patients with central neurological complications have high morbidity and mortality. Moreover, the clinical manifestations of these patients are diverse. Therefore, clinicians should examine the medical history, perform physical examination, and complete auxiliary examination to identify SFTS patients complicated with CNS damage in a timely manner. More particularly, serum SFTSV RNA, lactate dehydrogenase levels, pulmonary rales, and atrial fibrillation should be monitored in such patients.

## Data availability statement

The original contributions presented in the study are included in the article/Supplementary material, further inquiries can be directed to the corresponding authors.

## Ethics statement

The studies involving human participants were reviewed and approved by the Ethics Committee of the First Affiliated Hospital of Nanjing Medical University (Jiangsu Provincial People's Hospital). Written informed consent for participation was not required for this study in accordance with the national legislation and the institutional requirements.

## Author contributions

MW originally drafted and edited the manuscript. PH, WL, and WT did statistical analysis. TC and TZ performed the experiments. JS and HX contributed to literature search. CZ and JL recruited patients. MY designed, reviewed, and revised the manuscript. All authors contributed to the article and approved the submitted version.

## Funding

This work was supported by Open Research Fund Program of the State Key Laboratory of State Key Laboratory of Pathogen and Biosecurity (No. SKLPBS2137), Science Foundation for Distinguished Young Scholars of Jiangsu Province (BK20190106), the Social Development Fund of Jiangsu Province (No. BE2022682), Natural Science Foundation of Jiangsu Province (BK20221196), Key Project of Natural Science Foundation of Yunnan Province (2019FA005), Science and Technology Plan of Hainan Province (Clinical Research Center) (LCYX202103 and LCYX202204), Hainan Province Science and Technology Special Fund (ZDYF2022SHFZ067), Priority Academic Program Development of Jiangsu Higher Education Institutions (PAPD), and Clinical Research Center for Emerging Respiratory infectious diseases (HS2020002).

## Conflict of interest

The authors declare that the research was conducted in the absence of any commercial or financial relationships that could be construed as a potential conflict of interest.

## Publisher’s note

All claims expressed in this article are solely those of the authors and do not necessarily represent those of their affiliated organizations, or those of the publisher, the editors and the reviewers. Any product that may be evaluated in this article, or claim that may be made by its manufacturer, is not guaranteed or endorsed by the publisher.

## Abbreviations

SFTS: severe fever with thrombocytopenia syndrome
SFTSV: severe fever with thrombocytopenia syndrome virus
PLT: platelet
WBC: white blood cell
MCP-1: monocyte chemoattractant protein-1
IL: interleukin
IFN-γ: interferon-γ
CNS: central nervous system
PT: prothrombin time
APTT: activated partial thromboplastin time
TT: thrombin time
ALT: alanine aminotransferase
AST: aspartate aminotransferase
BUN: blood urea nitrogen
Cr: creatinine
LDH: lactate dehydrogenase
CK: creatine kinase
α-HBDH: α-hydroxybutyrate dehydrogenase
PCR: polymerase chain reaction
