# Supplementary material for: Risk factors of severe fever with thrombocytopenia syndrome combined with central neurological complications: A five-year retrospective case–control study
Source: Front Microbiol. 2022 Nov 3;13:1033946. doi: 10.3389/fmicb.2022.1033946 (PMC9668900; doi:10.3389/fmicb.2022.1033946)
Supplement: Supplementary file 1 [file Data_Sheet_1.docx]

Supplementary Table S1. Comparison of laboratory parameters between the two groups during the multiple organ dysfunction stage.

|  | CNS (n=64*) | N (n=74*) | statistic | *p* value |
| --- | --- | --- | --- | --- |
| WBC ^a^(×10^9/L) | 3.94(2.21,5.90) | 3.18(2.44,4.14) | -1.985 | 0.047 |
| PLT ^a^(×10^9/L) | 32(23,46) | 47(30,77) | -3.110 | 0.002 |
| PT ^a^(s) | 12.7(11.7,14.2) | 11.6(10.9,12.4) | -4.908 | <0.001 |
| APTT ^a^(s) | 46.7(39.3,61.0) | 35.0(30.0,44.4) | -5.269 | <0.001 |
| TT ^a^(s) | 34.5(23.2,120.0) | 21.7(18.8,25.9) | -5.151 | <0.001 |
| D-Dimer ^a^(mg/L) | 2.11(1.49,3.83) | 1.19(0.73,2.16) | -4.166 | <0.001 |
| ALT ^a^(U/L) | 132.9(81.2,207.7) | 103.6(67.8,164.7) | -1.753 | 0.080 |
| AST ^a^(U/L) | 399.2(223.2,787.1) | 187.6(107.6,311.0) | -5.222 | <0.001 |
| BUN ^a^(mmol/L) | 9.5(5.6,14.2) | 5.3(3.9,6.7) | -5.461 | <0.001 |
| Cr ^a^(μmol/L) | 74.3(58.1,171.8) | 57.2(49.6,70.0) | -4.287 | <0.001 |
| LDH ^a^(U/L) | 1611.5(1096.8,1611.5) | 643.0(393.0,1148.0) | -6.442 | <0.001 |
| CK ^a^(U/L) | 867.0(398.5,1726.3) | 262.0(85.5,734.5) | -5.106 | <0.001 |
| α-HBDH ^a^(U/L) | 741.5(480.0,1216.0) | 357.0(255.0,511.0) | -6.211 | <0.001 |
| K ^a^(mmol/L) | 3.28(3.07,3.77) | 3.32(3.05,3.60) | -0.989 | 0.323 |
| Na ^a^(mmol/L) | 135.1(131.5,138.1) | 136.3(133.0,138.9) | -0.171 | 0.865 |
| Ca ^a^(mmol/L) | 1.91(1.81,2.03) | 1.98(1.87,2.09) | -1.462 | 0.144 |

**Note:** ^a^ By means of the nonparametric test, expressed as *M* (*Q25, Q75)*, *M* is the median, *Q25* is the lower quartile, *Q75* is the upper quartile. *During the multiple organ dysfunction stage, because some indicators (including PT, APTT, TT, LDH, CK, α-HBDH, etc.) were not reexamined due to the death or improvement of some patients, and SFTSV RNA was also not reexamined in majority of the surviving patients, only 64 cases in the case group and 74 cases in the control group were finally enrolled, and SFTSV RNA was not included in the analysis.

**Abbreviations:** CNS, the case group; N, the control group; WBC, white blood cell; PLT, platelet; PT, prothrombin time; APTT, activated partial thromboplastin time; TT, thrombin time; ALT, alanine aminotransferase; AST, aspartate aminotransferase; BUN, blood urea nitrogen; Cr, creatinine; LDH, lactate dehydrogenase; CK, creatine kinase; α-HBDH, α-hydroxybutyrate dehydrogenase.

Supplementary Table S2. Comparison of serum cytokines between the two groups during the fever stage.

|  | IL-2 (pg/mL) | IL-4 (pg/mL) | IL-6 (pg/mL) | IL-10 (pg/mL) | TNF (pg/mL) | IFN-γ (pg/mL) |
| --- | --- | --- | --- | --- | --- | --- |
| Normal range | 0-5.71 | 0-3.00 | 0-5.30 | 0-4.91 | 0-4.60 | 0-7.42 |
| CNS (n=3) | Negative | Negative | 13.91 | 9.65 | Negative | Negative |
|  | Negative | Negative | 697.95 | 27.28 | Negative | 45.88 |
|  | Negative | Negative | 72.75 | 103.57 | Negative | 14.06 |
| N (n=2) | Negative | Negative | 36.67 | Negative | 5.45 | Negative |
|  | Negative | Negative | Negative | Negative | Negative | Negative |

**Abbreviations:** CNS, the case group; N, the control group; IL: interleukin; TNF: tumor necrosis factor; IFN-γ: interferon-γ.

Supplementary Table S3. Comparison of serum cytokines between the two groups during the multiple organ dysfunction stage.

|  | IL-2 (pg/mL) | IL-4 (pg/mL) | IL-6 (pg/mL) | IL-10 (pg/mL) | TNF (pg/mL) | IFN-γ (pg/mL) |
| --- | --- | --- | --- | --- | --- | --- |
| Normal range | 0-5.71 | 0-3.00 | 0-5.30 | 0-4.91 | 0-4.60 | 0-7.42 |
| CNS (n=10) | Negative | Negative | 32.8 | 8.54 | 29.07 | Negative |
|  | Negative | Negative | 670.88 | 158.6 | 4.61 | Negative |
|  | Negative | Negative | 32.8 | 78.08 | Negative | Negative |
|  | Negative | Negative | 16840.83 | 53.73 | 79.68 | Negative |
|  | Negative | Negative | 43.23 | 5.03 | Negative | Negative |
|  | Negative | Negative | 422.26 | 49.63 | Negative | Negative |
|  | Negative | Negative | 79.86 | 68.08 | Negative | Negative |
|  | Negative | Negative | 697.37 | 51.55 | Negative | Negative |
|  | Negative | Negative | 17.19 | 3.07 | Negative | Negative |
|  | Negative | Negative | 58.63 | 76.06 | Negative | Negative |
| N (n=11) | Negative | Negative | 988.35 | 0.34 | 21.95 | Negative |
|  | Negative | Negative | 3.24 | 4.82 | Negative | Negative |
|  | Negative | Negative | 7.98 | 13.68 | Negative | Negative |
|  | Negative | Negative | 123.77 | 11.12 | Negative | Negative |
|  | Negative | Negative | 26.12 | 1.58 | Negative | Negative |
|  | Negative | Negative | 14.82 | 21.65 | Negative | Negative |
|  | Negative | Negative | 638.54 | 4.75 | 17.96 | Negative |
|  | Negative | Negative | 35.72 | 34.01 | Negative | Negative |
|  | Negative | Negative | 1466.35 | 3.03 | 11.17 | Negative |
|  | Negative | Negative | 775.26 | 1.1 | Negative | Negative |
|  | Negative | Negative | 18.01 | 9.63 | Negative | Negative |
| statistic | - | - | -0.704 | -2.676 | - | - |
| *p* value | - | - | 0.481^a^ | **0.007**^a^ | - | - |

**Note:** ^a^ By means of the nonparametric test; “-”: No relevant data.

**Abbreviations:** CNS, the case group; N, the control group; IL: Interleukin; TNF: tumor necrosis factor; IFN-γ: interferon-γ.
